# Comparison on genomic prediction using pedigree BLUP and single step GBLUP through the Hanwoo full-sib family

**DOI:** 10.5713/ab.22.0327

**Published:** 2023-05-02

**Authors:** Eun-Ho Kim, Ho-Chan Kang, Cheol-Hyun Myung, Ji-Yeong Kim, Du-Won Sun, Doo-Ho Lee, Seung-Hwan Lee, Hyun-Tae Lim

**Affiliations:** 1 Department of Animal Science, Gyeongsang National University, Jinju 52828, Korea; 2 Department of Animal Science and Biotechnology, Gyeongsang National University, Jinju 52828, Korea; 3 Gyeongnam Animal Science and Technology, Gyeongsang National University, Jinju 52828, Korea; 4 Department of Animal Science and Biotechnology, Chungnam National University, Daejeon 34134, Korea

**Keywords:** Acuuracy, Full-sib, Genomic Selection, Genotype, Hanwoo

## Abstract

**Objective:**

When evaluating individuals with the same parent and no phenotype by pedigree best linear unbiased prediction (BLUP), it is difficult to explain carcass grade difference and select individuals because they have the same value in pedigree BLUP (PBLUP). However, single step GBLUP (ssGBLUP), which can estimate the breeding value suitable for the individual by adding genotype, is more accurate than the existing method.

**Methods:**

The breeding value and accuracy were estimated with pedigree BLUP and ssGBLUP using pedigree and genotype of 408 Hanwoo cattle from 16 families with the same parent among siblings produced by fertilized egg transplantation. A total of 14,225 Hanwoo cattle with pedigree, genotype and phenotype were used as the reference population. PBLUP obtained estimated breeding value (EBV) using the pedigree of the test and reference populations, and ssGBLUP obtained genomic EBV (GEBV) after constructing and H-matrix by integrating the pedigree and genotype of the test and reference populations.

**Results:**

For all traits, the accuracy of GEBV using ssGBLUP is 0.18 to 0.20 higher than the accuracy of EBV obtained with PBLUP. Comparison of EBV and GEBV of individuals without phenotype, since the value of EBV is estimated based on expected values of alleles passed down from common ancestors. It does not take Mendelian sampling into consideration, so the EBV of all individuals within the same family is estimated to be the same value. However, GEBV makes estimating true kinship coefficient based on different genotypes of individuals possible, so GEBV that corresponds to each individual is estimated rather than a uniform GEBV for each individual.

**Conclusion:**

Since Hanwoo cows bred through embryo transfer have a high possibility of having the same parent, if ssGBLUP after adding genotype is used, estimating true kinship coefficient corresponding to each individual becomes possible, allowing for more accurate estimation of breeding value.

## INTRODUCTION

Genetic improvement of domestic animals has developed from a method where animals with good records in phenotype are selected into genetics evaluation methods that utilize all information on the phenotype and pedigree that a certain animal possesses. Best linear unbiased prediction (BLUP) which calculate estimated breeding value (EBV) by correcting the environmental elements that affect the pedigree and phenotype of offspring and siblings of individual animals in question has had a great impact on genetic improvement of livestock [1]. Korean beef (Hanwoo) an important Korean livestock product has been successfully genetically improved by applying such methods to bulls, but it has been difficult to evaluate the ability and genetically improve cows due to the small number of offspring as they can only give birth once a year through natural breeding or artificial insemination. To solve this problem of information insufficiency, the method of ovum pick up has been introduced to maximize the productivity of cows. This technique uses ultrasound to collect immature ova from the ovaries of living cows, mature those ova outside the animals’ body a process called *in vitro* maturation and then form fertilized eggs using sperm. In this technique, 11.9 ova can be collected and 3.6 fertilized eggs can be produced annually from one cow, allowing livestock geneticists to secure large amounts of phenotypic information on descendant animals in a short period of time [2]. However, in the case of cows bred through embryo transfer, the probability of them having full sibs from the same parents is high. When genetic evaluation is performed through pedigree, test individuals, whose phenotype is not known until slaughter, Mendelian sampling effect is not considered at all. As a result, the probability of them having common genes due to identical by descent, causing a problem in that each individual in the family are estimated to have the same EBV [3,4]. Also, Sullivan [5] reported that the evaluation of EBV without taking Mendelian sampling into consideration results in biased information, and if this problem continues, the effectiveness of genetic improvement decreases and the frequency of unmasked deleterious genetic mutations will increase.

Genome selection, which has been widely used in the livestock industry, recently has had a positive influence on animal breeding through its advantages such as estimation with higher accuracy than traditional breeding methods [6]. Genome selection is a method where genomic EBV (GEBV) is estimated by using the phenotype and genotype of single nucleotide polymorphism (SNP) densely distributed on chromosomes. It can estimate with much higher accuracy than with pedigrees by estimating a kinship coefficient that is closer to the real value of the individual, using the ratio of genetic mutations and gene effects between different individuals [4,7]. By applying this genome selection method to full-sib groups through embryo transfer, estimation of breeding value suitable to each individual becomes possible, so it is thought that individual selection based on higher accuracy than before can be carried out. As such, this study estimates breeding value and accuracy with pedigree BLUP (PBLUP) and single step genomic BLUP (ssGBLUP) using pedigree and genotype of full-sib family by embryo transfer, and the results have been compared.

## MATERIALS AND METHODS

All animal care and treatment procedures were conducted in strict accordance with the Animal Ethics Committee of Gyeongsang National University, Korea, and performed in accordance with the Committee's guidelines and regulations (Approval No.: GNU-220825-A0094).

### Animal population and phenotype

The test population used in this study consisted of a total of 467 Hanwoo cattle from 18 families, provided with individual identification numbers of Hanwoo produced by embryo transfer from GAST, Gyeongsang national university. Among the families with the same parents, 408 Hanwoo cattles from 16 families were finally selected by excluding less than 5 slaughtered animals and those with abnormal carcass grades. The reference population used for the analysis was provided with pedigree, genotype, and phenotype from the BioGreen 21 program (Molecular breeding Program) of National Institute of Animal Science (NIAS), Rural Development Administration (RDA), South Korea. Through the normality analysis, individuals with a slaughter age of less than 26 months or more than 36 months and individuals with abnormalities in carcass grade were found and removed. Finally, 14,225 head were used for the analysis.

The phenotype shared by the two reference population involved following the Livestock Grade Determination Standard Detail #2014-4 posted by the Ministry of Agriculture, Food and Rural Affairs (MAFRA), with carcass traits measured 24 hour of refrigeration after the butchery. Carcass weight (CWT) measured as the sum of left and right frozen body weights, while eye muscle area (EMA) was measured by cutting the area between the left and right thoracic vertebrae and first lumbar perpendicular to the vertebra and measuring the area of the last thoracic vertebrae. Back-fat thickness (BFT) was measured by the area that is 2/3 inwards towards the belly along with the EMA. And marbling score (MS) was visually measured by comparing the degree of fat deposit in the muscles of the EMA measurement area with the standard table (1 = devoid and 9 = abundant).

### Animal pedigree

To collect the pedigree to be used in BLUP, the individual identification numbers were searched for pedigree at Korea Animal Improvement Association (KAIA) to establish a pedigree tree through whole backtracking, and renumbering took place to arrange into Animal, Sire and Dam. After that, outliers were corrected using the R Software [8] suitable for large-scale information processing and was combined with the acquired pedigree tree of test and reference population. The constructed pedigree tree was 761 Hanwoo cattle in the test population and 58,669 Hanwoo cattle in the reference population. By combining the two pedigree trees, duplicated individuals were removed, and a total 59,141 Hanwoo cattle were finally used for analysis.

### Genotype, quality control and imputation

To collect the genotype to be used for ssGBLUP, high purity genomic DNA was acquired by extracting it from the hair and blood, then genotype was collected using Hanwoo 50K SNP BeadChip Ver.1 and Bovine 50K SNP BeadChip Ver. 2, Ver. 3 (Illumina Inc., San Diego, CA, USA). The acquired large-scale genotype was converted to a form suitable for PLINK1.9 [9] using GenomoStudio 2.0 (Illumina Inc., San Diego, CA, USA), and the missing genotypes underwent imputation of reference population genotype using Eagle Ver. 2.4.1 [10] and Minimac3 Ver. 2.0.1 [11]. Afterward, the genotype of test and reference population were combined into common SNP information, and for Quality Control, PLINK1.9 [9] was used to select SNP with less than 1% minor allele frequency, 10% or more missing genotype, and less than 10^−6^ Hardy-Weinberg equilibrium, finally using a total of 41,564 SNP markers.

### Estimated of estimated breeding value by pedigree best linear unbiased prediction

The EBV, prediction error variance (PEV) and genetic parameters for each trait were estimated by applying the numerator relationship matrix (NRM) constructed with pedigree in the BLUPF90 program [12]. For the fixed effect, the birth year, birth month, age at slaughter, slaughter place were used, and the mixed model equation is as follows:


$$\begin{array}{l} \text{Y}=X\beta +Zu+e\\ \text{Var }\left(\frac{u}{e}\right)=\left(\begin{array}{cc} A\sigma_{\alpha }^{2} & 0\\ 0 & I\sigma_{e}^{2} \end{array}\right) \end{array}$$

Here, *Y* is the vector for the observed value of economic traits, *X* is the vector for the fixed effect and *β* is the vector for the estimated value of the fixed effects. *Z* is the vector for the random effects of individuals and u is the vector for the estimated value of individuals and e is the vector for random errors. E(y) = Xβ, $\text{Var}(\text{u})=\text{G}=\text{A}\sigma_{\alpha }^{2}$, Cov(μ,e) = 0 is assumed to give Var(y) = V = ZGZ′ + R. Here, A is the NMR constructed by the pedigree between individuals, $\sigma_{\sigma }^{2}$ is the additive genetic variance and $\sigma_{e}^{2}$ is the random environmental variance. Heritability was calculated $h^{2}=\sigma_{\alpha }^{2}/\sigma_{p}^{2}$, where *h*^2^ is heritability, $\sigma_{p}^{2}$ was $\sigma_{\alpha }^{2}+\sigma_{e}^{2}$.

### Estimated of genomic estimated breeding value by single step genomic best linear unbiased prediction

While ssGBLUP and PBLUP used the same fixed effect, GRM was established using genotype and NRM that contain the relationship coefficient between individuals to perform the GEBV estimation. Instead of A^−1^, GBLUP uses H^−1^ inverse matrix of H that is based on pedigree and genotype, to estimate GEBV, and the mixed model equation is as follows:


$$\begin{array}{l} \left[\begin{array}{cc} X^{\prime }R^{-1}X & X^{\prime }R^{-1}Z\\ Z^{\prime }R^{-1}X & Z^{\prime }Z+\alpha H^{-1} \end{array}\right]\;\left[\begin{array}{c} \hat{b}\\ \hat{u} \end{array}\right]=\left[\begin{array}{c} X^{\prime }R^{-1}y\\ Z^{\prime }R^{-1}y \end{array}\right]\\ H^{-1}=A^{-1}+\left[\begin{array}{cc} 0 & 0\\ 0 & G^{-1}+A_{22}^{-1} \end{array}\right] \end{array}$$

Here, H is relational matrix based on pedigree and genotype; A is NRM constructed using pedigree, G is GRM constructed using genotype, α is the additive genetic variance of the individual, with R is the variation matrix on residual effect. Especially, G was established using the following relation formula [13].


$$\text{G}=\frac{ZZ^{\prime }}{2\;\Sigma \;p_{j}(1-p_{j})}$$

Here, Z = M-P, where M is n (number of individual) X m (number of SNP), which is a matrix when converting homozygote (AA, BB), heterozygote (AB), each into −1,0,1. And P is the frequency (pj) of second allele on the jth locus. Defining row j of matrix p as 2(pj −0.5), p becomes the frequency value of expected genotype that averages value of 0.

### Estimated accuracy

The PEV obtained from PBLUP and ssGBLUP was used to estimate the accuracy EBV and GEBV for each trait using the equation below.


$$\text{Acc}=\sqrt{1-(PEV/\sigma_{\alpha }^{2})}$$

Here, Acc is accuracy of the EBV and GEBV. PEV is the prediction error variance of the EBV and GEBV, and $\sigma_{\alpha }^{2}$ is the additive genetic variance. PEV is the error deviation range of the EBV and GEBV estimated for each individual. It is the diagonal element of the inverse left side of the mixed model equation of each analysis method, while can be earned using REMLF90 of the BLUPF90 package.

## RESULTS

### Basic statistical analysis of the test and reference population

The phenotype of the test and reference population used for the analysis are CWT, EMA, BFT and MS, and the basic statistics are shown in Table 1. The average and standard deviation of each trait were 475.6±70.8 kg, 102.4±16.8 cm^2^, 12.7±4.9 mm, and 6.5±2.0 point in test population, 441.1±47.6 kg, 95.8±11.4 cm^2^, 13.9±4.2 mm, 5.9±1.8 Point in the reference population. The average value of all traits was high in the test population. Looking at the coefficient of variation of the two populations, BFT and MS were significantly higher, indicating that the two traits had a larger deviation than the other two traits.

### Comparison of estimated results with PBLUP and ssGBLUP

The estimated genetic parameters according to the analysis method was shown in Table 2 with values estimated by genetic variance ( $\sigma_{a}^{2}$), residual variance ( $\sigma_{e}^{2}$), phenotypic variance ( $\sigma_{p}^{2}$), heritability and standard error (SE), accuracy and standard deviation. In the order of CWT, EMA, BFT, and MS, the heritability estimated by PBLUP was 0.29±0.03, 0.29±0.03, 0.23±0.03, and 0.39±0.04, and the heritability estimated by ssGBLUP was 0.43±0.01, 0.39±0.01, 0.36±0.01, and 0.47±0.01, indicating the ssGBLUP was high. The formula for calculating heritability is $h^{2}=\sigma_{a}^{2}/(\sigma_{a}^{2}+\sigma_{e}^{2}=\sigma_{p}^{2})$. The phenotypic variance estimated by the two analysis methods is similar, but the difference is due to the relatively high genetic variance estimated by ssGBLUP. The SE of heritability estimated in each analysis method were 0.03, 0.03, 0.03, and 0.04 in PBLUP, and ssGBLUP was 0.01 in all traits, which was lower than the SE estimated in PBLUP. Looking at the estimated accuracy, the average accuracy of PBLUP was 0.53 ±0.02, 0.53±0.02, 0.52±0.02, and 0.54±0.02, and the accuracy of ssGBLUP was 0.73±0.04, 0.71±0.04, 0.70±0.04, and 0.74 ±0.04 in the order of CWT, EMA, BFT, and MS. The difference in the estimated accuracy according to the analysis method was 0.18 to 0.20, and the accuracy estimated by ssGBLUP was high.

CWT, EMA, BFT, and MS used in the analysis were multiple traits of animal model according to the analysis method, and the genetic and phenotypic correlation between each trait were estimated and shown in Table 3. As result, the genetic correlation range was 0.05±0.09 to 0.58±0.06 in PBLUP and 0.13±0.03 to 0.50±0.02 in ssGBLUP, and the phenotypic correlation range was 0.12±0.01 to 0.50±0.01 in PBLUP and 0.17±0.01 to 0.51±0.01 in ssGBLUP. The values of genetic and phenotypic correlations estimated by the two analysis methods were similar. The genetic correlations of EMA and BFT were negatively correlated with −0.05 in PBLUP and −0.13 in ssGBLUP, but other traits had positive correlations. In addition, the SE of phenotypic correlations estimated in each analysis method were all 0.01, showing the same value, but the SE of genetic correlations were 0.06 to 0.09 in PBLUP and 0.02 to 0.03 in ssGBLUP, which was lower than the SE estimated in PBLUP.

The EBV and GEBV, average value and standard deviation of carcass grade estimated according to analysis method have been categorized by family in Table 4. Families that had a large gap between EBV and GEBV among the CWT included number 11, which had a weight of 22.11 kg, number 8, which had a EMA of 5.06 cm^2^, number 9, which had a BFT of 1.88 mm, number 17, which had a MS of 1.67 point, and other families had different estimated value. The difference between EBVs obtained from both analysis methods was that standard deviation of EBV did not exist, but this alone is not enough to explain the various phenotype as all individuals within the same family are estimated to have the same EBV. On the other hand, standard deviation exists for GEBV, so it can be assumed that different GEBV have been estimated for each individual. If the carcass grade and GEBV for each parent combination is looked into, the family with the best grade in CWT and EMA is number 11. The family with the best grade for BFT is number 9. And MS is number 15 for GEBV, and number 3 for overall highest carcass grade MS. Families with high scores in all aspects all used Sire 3, family numbers 1 and 8 had half-sib relationships by Donor for Dam 1, and family numbers 10 and 14 for Dam 9, and the GEBV and carcass grades of both populations turned out to be similar. On the other hand, family numbers 2 and 7 had half-sib relationships from their father in Sire 2, and they had major differences in carcass grade of CWT and EMA, and in Sire 1, families number 1 and 3 had different carcass grades in all categories apart from BFT. Through these data, it was found that there were differences in carcass grade by combination of each father and mother.

**Figure 1:**
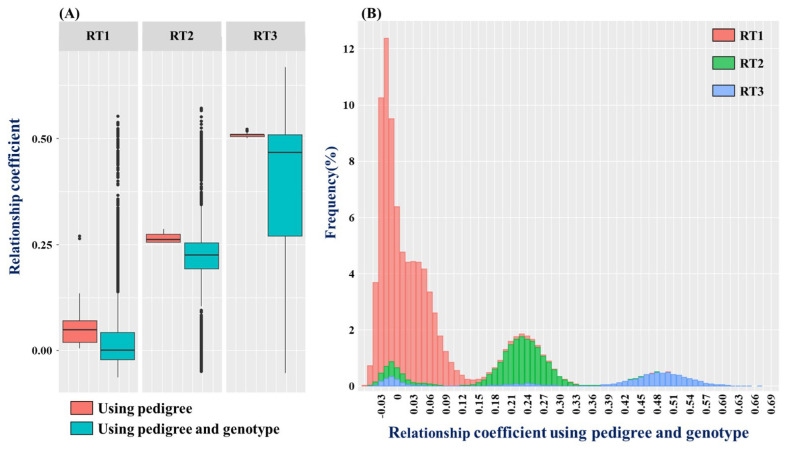
Estimated kinship coefficient using the pedigree and genotype of the test population. (A) is a boxplot showing the kinship relationship matrix diagram used for PBLUP and ssGBLUP by dividing it into low relatedness population, half-sib population, full-sib population. (B) shows the kinship coefficient matrix diagram used for ssGBLUP by frequency by population (RT1, Low relatedness population; RT2, Half-sib population; RT3, Full-sib population).

The test population is divided up into low relatedness population (RT1), a half-sib family where only one side of the parents is the same (RT2), and a full-sib family that has the same parents (RT3) and the relationship graph used to analyze the kinship coefficient distribution of each population is shown as a boxplot and histogram in Figure 1A. It shows each population according to analysis method in boxplots, the kinship coefficient average was 0.0536, 0.2645, and 0.5083 for RT1, RT2, and RT3, respectfully, and for ssGBLUP, the numbers were 0.0191, 0.2048, 0.3803, respectfully, showing that the kinship coefficient numbers for the PBLUP method was higher. However, if the range of estimated kinship coefficient is examined, the kinship coefficient range for PBLUP was 0.0045 to 0.2707, 0.2538 to 0.2860, and 0.5005 to 0.5225, −0.0628 to 0.5527, −0.0494 to 0.5711, −0.0526 to 0.6684 for ssGBLUP, showing that the kinship coefficient range for ssGBLUP was much wider. There are a lot of off-diagonal elements that diverged from the boxplot distribution range for ssGBLUP, which is due to the elements being far from the average and having a low frequency, and this tendency appeared in Figure 1B, which shows the frequency of blood relationship used in ssGBLUP. The total range of blood relationship used in ssGLBUP is −0.0628 to 0.6684, which is very wide. RT1, RT2, and RT3 are distributed widely, but RT1 is skewed to the average value, and RT2 and RT3 show a normal distribution curve with a gentle slope. All populations show an off-diagonal element with negative value around the minimum value, which is caused during the process of estimating kinship coefficient using the SNP marker’s minor allele frequency (MAF) value, and it does not pose a major problem when estimating the genome breeding value with the negative value set up as mixed model equation [14].

## DISCUSSION

### The importance of basic statistics

In order to increase the effectiveness of livestock genetic improvement, it is important to form and select population s with good carcass grades. Lee et al [15] collected carcass grade data of 12,000 cows from a reference population to estimate the GEBV of cows raised in the Gyeongi-do Province area, and results showed that CWT, EMA, BFT, and MS were 441.21±51.53 kg, 95.92±12.10 cm^2^, 14.41±4.87 mm, 6.10± 1.84 point, respectfully. Lee et al [16] collected carcass grade data of steers raised in Gangwon-do Province farms, and data of CWT, EMA, BFT, and MS were 431.77±51.43 kg, 91.22±10.75 cm^2^, 13.30±5.14 mm, 5.66±1.88 point, respectfully, which matched the numbers of the two earlier studies. The average carcass grade of the top 10% of market price among Hawnoo in 2021 was 449.9±56.1 kg, 106.6±13.10 cm^2^, 12.40±4.1 mm, and 8.40±0.9 point, which is similar to the data of the test population apart from MS [17]. The test population can be part of the top 10% of Hanwoo, and if it is used for livestock improvement selection, it is thought that the production of individuals with higher carcass grades is possible (Table 1). In the study’s statistical analysis results, the variation factor of BFT and MS were higher than that of other traits, and this is thought to be the result of both traits having wide deviations by the part that is measured, which is not the case in other traits. Choi et al [18] also show that the variation factor of BFT and MS of cows that have undergone progeny tests is three to four times higher than that of other traits, and this matches the results of this research study.

### Comparison of estimated results with PBLUP and ssGBLUP

In the preceding research study done on genetic parameters estimated using both analysis methods, the research of Cesarani et al [19] compares the heritability of fatty acids using Sarda breed cows’ pedigree and genotype, and results show that heritability estimated with pedigree is lower than estimated using ssGLBUP. And the research of Esfandyari et al [20] compares the heritability and genetic correlation of growth traits and carcass traits using pedigree and genotype of pigs with pure and mixed bred, and results show that in all traits, heritability estimated using ssGBLUP is higher, and genetic correlation estimated through both analysis methods is similar. Also, both preceding research studies reported that estimated standard deviation is lower when using ssGBLUP than when using PBLUP, and this result corresponds with the this study’s results (Tables 2, 3). In fact, many studies that shown that including genotype when estimating genetic parameters is more accurate than using pedigree in many different cattle breeds [21–23].

Differences in genetic parameters estimated using PBLUP and ssGBLUP are based on fundamental differences in genetic models formed upon coefficient matrices used in each analysis method [24]. The simple formula for offspring’s true breeding value (TBV) is calculated by TBV_offspring_ = 1/2 TBV_sire_ + 1/2 TBV_dam_ + Mendelian sampling term, which means that an offspring’s genetic ability is determined by adding the genetic abilities passed on from the parents and the Mendelian sampling effect. Here, Mendelian sampling measures the genetic variability between siblings, relates to the effect of mixing up genes randomly passed on from both parents, and phenotypic characteristic, record, and other traits of each individual differ due to linkage disequilibrium and heterozygote fluctuation [25]. Therefore, when estimating the breeding value of an individual, accurate individual selection is only possible when ability evaluation that takes Mendelian sampling into consideration is carried out. NRM used in PBLUP is a matrix that estimates kinship coefficient based on pedigree, and is difficult to consider accurate Mendelian sampling as the values are estimated based on expected values of alleles shared between descendants from a common ancestor. Also, even if there are 2, 10, or 20 individuals within the same full-sib family with the same parents, the expected value of kinship coefficient of all individuals, whose without phenotype, is calculated as 0.5, so all of the family’s individuals are estimated to have the same EBV (Table 4). As such, genetic ability testing of full-sib families using PBLUP lacks reliability [26]. On the other hand, H-matrix used in ssGBLUP is a new kind of kinship coefficient matrix that combines NRM and GRM. Moreover, since it estimates kinship coefficient based on gene frequency shared by each individual, it has a high kinship coefficient and wide range even in half-sib families and families with low relationships as shown in Figure 1B, making it possible to identify the connecting points of a number of individuals. Also, through accurate coefficient estimation of gene mutation of each individual, accurate estimation of breeding value that takes Mendelian sampling into consideration becomes possible [25,27,28].

The accuracy of GEBV estimated using ssGLBUP (Table 2) shows that the estimation is higher than that of PBLUP, and as explained earlier, this is due to the difference in coefficient matrices, and using ssGBLUP which uses real kinship coefficient values can enable high-accuracy estimation as the method estimates more connections between individuals than PBLUP and decreases residual variance [29].

After, In order to increase the accuracy of genetic ability evaluation of the test population, measures such as a reference population that has many relationship connections and has various genetic mutations, density of SNP Chip, and accurate pedigree tree is necessary. Among these factors, the most influential one is the number of offspring from the individual to be tested. The higher the number of offspring, the lower the deviation that estimates the genetic mutation of individuals to be tested, which means lower standard deviation and higher accuracy [30]. If the number of offspring is increased through embryo transplant, the GEBV accuracy of not only the offspring and sib test of the father but also those of the mother will increase. And a suitable selection criterion that takes into consideration the order of priority, how closely related individuals are, and the direction of genetic improvement according to the combination of parents can be established (Table 4). Also, if the genotype of offspring cumulates, genotype of half-sib families will increase, allowing for accurate evaluation of genetic ability and increase in effectiveness of genetic improvement.

## CONCLUSION

This research study uses pedigree and genotype of full-sib families, PBLUP, ssGBLUP to estimate accuracy and breeding value, and using the results estimated from both analysis methods, an effective method of analysis for full-sib families has been found. The accuracy of GEBV using ssGBLUP is 0.18 to 0.20 higher than the accuracy of EBV obtained with PBLUP. Since the value of EBV is estimated based on expected values of alleles passed down from common ancestors. It does not take Mendelian sampling into consideration, so the EBV of all individuals within the same family is estimated to be the same value, but GEBV makes estimating true kinship coefficient based on different genotypes of individuals possible, so GEBV that corresponds to each individual is estimated rather than a uniform GEBV for each individual. Since Hanwoo cows bred through embryo transfer have a high possibility of having the same parent, if ssGBLUP which estimates GEBV after adding genotype is used, estimating true kinship coefficient corresponding to each individual becomes possible, allowing for accurate estimation of breeding value. Also, if embryo transplant which shows high efficiency at a short period of time in terms of productivity and genetic improvement is applied to individuals with high carcass grades, the effectiveness of genetic improvement can be maximized through high selection intensity.

## Figures and Tables

**Table 1:** Basic statistics of test and reference population used in the analysis

Type	No. animal	Trait	Mean	SD	Min	Max	CV (%)
Test population	408	CWT (kg)	475.6	70.8	166	655	14.9
		EMA (cm^2^)	102.4	16.8	30	143	16.4
		BFT (mm)	12.7	4.9	1	31	38.5
		MS (point)	6.5	2.0	1	9	30.5
Reference population	14,225	CWT (kg)	441.1	47.6	299	584	10.8
		EMA (cm^2^)	95.8	11.4	62	129	11.9
		BFT (mm)	13.9	4.2	1	26	30.6
		MS (point)	5.9	1.8	1	9	30.9

SD, standard deviation; CV, coefficient of variation; CWT, carcass weight; EMA, eye muscle area; BFT, back fat thickness; MS, marbling score.

**Table 2:** Estimated genetic parameters and heritability according to the analysis method

Method	Parameter	CWT	EMA	BFT	MS
PBLUP	$\sigma_{a}^{2}$	603.79	34.29	4.07	1.25
	$\sigma_{e}^{2}$	1,455.60	85.70	13.74	1.98
	$\sigma_{p}^{2}$	2,059.39	119.99	17.82	3.23
	*h*^2^±SE	0.29±0.03	0.29±0.03	0.23±0.03	0.39±0.04
	Accuracy±SD	0.53±0.02	0.53±0.02	0.52±0.02	0.54±0.02
ssGBLUP	$\sigma_{a}^{2}$	859.69	43.99	6.39	1.51
	$\sigma_{e}^{2}$	1,143.70	74.43	11.53	1.70
	$\sigma_{p}^{2}$	2,003.39	118.42	17.92	3.21
	*h*^2^±SE	0.43±0.01	0.37±0.01	0.36±0.01	0.47±0.01
	Accuracy±SD	0.73±0.04	0.71±0.04	0.70±0.04	0.74±0.04

CWT, carcass weight; EMA, eye muscle area; BFT, back fat thickness; MS, marbling score; PBLUP, best linear unbiased prediction using pedigree; ssGBLUP, best linear unbiased prediction using pedigree and genotype; $\sigma_{a}^{2}$, additive genetic variance; $\sigma_{e}^{2}$, residual variance; $\sigma_{p}^{2}$, phenotypic variance; *h*^2^±SE, heritability and standard error; SD, standard deviation.

**Table 3:** Genetic correlation and phenotypic correlation according to analysis method^1)^

Method	Trait	CWT	EMA	BFT	MS
PBLUP	CWT	-	0.50±0.07	0.23±0.09	0.29±0.08
	EMA	0.50±0.01	-	−0.05±0.09	0.58±0.06
	BFT	0.40±0.01	0.12±0.01	-	0.11±0.09
	MS	0.14±0.01	0.38±0.01	0.12±0.01	-
ssGBLUP	CWT	-	0.47±0.02	0.22±0.03	0.16±0.03
	EMA	0.51±0.01	-	−0.13±0.03	0.50±0.02
	BFT	0.47±0.01	0.20±0.01	-	0.03±0.03
	MS	0.21±0.01	0.40±0.01	0.17±0.01	-

CWT, carcass weight; EMA, eye muscle area; BFT, back fat thickness; MS, marbling score; PBLUP, best linear unbiased prediction using pedigree; ssGBLUP, best linear unbiased prediction using pedigree and genotype.

1) Genetic correlation (above diagonal) and phenotypic correlation (below diagonal) by trait according to the analysis method. And correlation values were shown as coefficients and standard errors.

**Table 4:** Comparison of GEBV and EBV, phenotype according to population^1)^

Pop	Parents	CWT					EMA				
	
		EBV (kg)	Acc_EBV_	GEBV (kg)	Acc_GEBV_	Phenotype (kg)	EBV (cm^2^)	Acc_EBV_	GEBV (cm^2^)	Acc_GEBV_	Phenotype (cm^2^)
1	Sire 1 X Dam 1	29.8	0.5	22.9±24.7	0.7	439.9±78.8	7.6	0.5	3.4±3.5	0.7	96.3±17.2
2	Sire 2 X Dam 2	16.4	0.6	1.1±17.9	0.7	433.7±80.8	8.7	0.6	4±3.5	0.7	94.8±24.7
3	Sire 1 X Dam 3	32.0	0.5	33.7±16.8	0.7	498±53.3	6.9	0.5	6.6±3.4	0.7	115.8±11.6
4	Sire 3 X Dam 4	48.1	0.5	35.5±12	0.7	510.7±40.4	9.5	0.5	7±3.1	0.7	101.4±12.3
5	Sire 3 X Dam 5	38.7	0.5	44.4±15.5	0.8	529.1±60.1	8.6	0.5	6.5±3.1	0.7	109.6±12.9
6	Sire 4 X Dam 6	14.3	0.5	−4±14	0.7	484.5±60.7	5.5	0.5	2.8±4.2	0.7	98.5±10.8
7	Sire 2 X Dam 7	5.4	0.5	3±19.4	0.7	475±41.2	6.2	0.5	5.5±3.4	0.7	100.8±9.7
8	Sire 4 X Dam 1	3.2	0.5	−8.8±21.4	0.7	437.3±68.9	2.3	0.5	−2.7±3.7	0.7	95.8±13.3
9	Sire 3 X Dam 8	36.6	0.5	38.8±15.7	0.7	490.8±41	10.3	0.5	12.4±2.8	0.7	116.5±11.2
10	Sire 5 X Dam 9	29.6	0.5	8.8±17.7	0.7	465.1±55.8	8.7	0.5	4.2±3.1	0.7	100±14.4
11	Sire 3 X Dam 10	48.8	0.5	70.9±19.3	0.7	565.6±48.5	10.9	0.5	13.9±2.9	0.7	119.3±14.6
12	Sire 3 X Dam 11	37.4	0.6	20.3±10.3	0.7	463±52.3	8.6	0.5	4±3.1	0.7	95.9±9.5
13	Sire 5 X Dam 12	20.3	0.5	12.8±23.6	0.7	442.9±46.5	7.9	0.5	6.2±4.7	0.7	100.6±12.6
14	Sire 3 X Dam 9	47.1	0.5	33.4±18.8	0.7	489.7±41.4	9.9	0.5	6.2±2.4	0.7	104.1±7.2
15	Sire 3 X Dam 13	42.5	0.5	44.6±12.9	0.7	521.9±42.2	9.2	0.5	12±2.9	0.7	106±11
16	Sire 4 X Dam 14	8.9	0.5	−8.1±22.1	0.7	468±22.4	3.3	0.5	0.5±2	0.7	95.2±7.6
Mean		28.4±13.8	0.5±0.0	21.5±27.4	0.7±0.0	475.6±70.8	7.9±2	0.5±0.0	5.5±5	0.7±0.0	102.4±16.8

		**BFT**					**MS**				
	
		**EBV (mm)**	**Acc** **_EBV_**	**GEBV (mm)**	**Acc** **_GEBV_**	**Phenotype (mm)**	**EBV (point)**	**Acc** **_EBV_**	**GEBV (point)**	**Acc** **_GEBV_**	**Phenotype (point)**

1	Sire 1 X Dam 1	−0.3	0.5	−1.3±0.9	0.7	11.4±3.7	1.2	0.5	1.2±0.6	0.7	6.6±2.2
2	Sire 2 X Dam 2	−0.8	0.5	0.1±1.2	0.7	13.3±5.5	1.0	0.6	0.2±0.7	0.7	5.4±2
3	Sire 1 X Dam 3	−0.2	0.5	−0.1±1.3	0.7	13.4±3.9	0.9	0.5	1.2±0.5	0.7	8.1±1.3
4	Sire 3 X Dam 4	−0.3	0.5	−1.1±1	0.7	11.7±3	1.3	0.5	1.3±0.6	0.7	7.2±1.5
5	Sire 3 X Dam 5	−0.8	0.5	0.1±1.3	0.7	12±4.7	1.1	0.5	1±0.6	0.8	6±1.8
6	Sire 4 X Dam 6	−0.4	0.5	−0.8±1.2	0.7	14.9±5.5	1.4	0.6	1.1±0.6	0.7	6.7±1.8
7	Sire 2 X Dam 7	−0.9	0.5	−1.6±0.8	0.7	13.8±5.1	0.5	0.5	−0.3±0.5	0.7	4.6±1.5
8	Sire 4 X Dam 1	0.0	0.5	−0.1±1.3	0.7	16.3±4.2	1.2	0.5	1.1±0.7	0.7	6.8±1.3
9	Sire 3 X Dam 8	−1.4	0.5	−3.2±1	0.7	7.1±2.8	1.4	0.5	1.4±0.6	0.8	7.9±1
10	Sire 5 X Dam 9	−0.8	0.5	−0.7±1.2	0.7	15.9±5.2	0.7	0.5	0.2±0.7	0.7	5.5±1.8
11	Sire 3 X Dam 10	−1.1	0.5	−1.6±1.1	0.7	11.8±3	1.1	0.5	1.3±0.5	0.8	7.3±1.1
12	Sire 3 X Dam 11	−1.1	0.5	−1±1.2	0.7	10.6±3.5	1.0	0.6	1±0.7	0.8	6.3±2.3
13	Sire 5 X Dam 12	0.1	0.5	−0.2±1	0.7	13.5±6.5	1.0	0.5	0.6±0.6	0.7	6.3±2.1
14	Sire 3 X Dam 9	−1.5	0.5	−0.7±1	0.7	14.8±4.1	1.1	0.5	0.5±0.5	0.7	5.9±1.6
15	Sire 3 X Dam 13	−0.6	0.5	−1±0.8	0.7	12.9±4.4	1.0	0.5	1.9±0.6	0.8	7.9±1.1
16	Sire 4 X Dam 14	−0.3	0.5	−0.9±0.5	0.7	11.4±1	0.9	0.5	0.3±1	0.7	6.2±0.4
Mean		−0.6±0.4	0.5±0.0	−0.9±1.4	0.7±0.0	12.7±4.9	1.1±0.2	0.5±0.0	0.9±0.8	0.7±0.0	6.5±2

CWT, carcass weight; EMA, eye muscle area; BFT, back fat thickness; MS, marbling score; EBV, estimated breeding value; AccEBV, accuracy of EBV; GEBV, genomic EBV; AccGEBV, accuracy of GEBV.

1) Shows the mean and standard deviation of breeding value and accuracy by population.
